# Quantum Effects in Evolution: Terrestrial Fine-Tuning of Magnetic Parameters

**DOI:** 10.34133/csbj.0125

**Published:** 2026-06-04

**Authors:** Betony Adams, Abbas Hassasfar, Ilya Sinayskiy, Alistair Nunn, Geoffrey Guy, Francesco Petruccione

**Affiliations:** ^1^ Department of Physics, Stellenbosch University, Stellenbosch 7600, South Africa.; ^2^ National Institute for Theoretical and Computational Sciences (NITheCS), Stellenbosch 7602, South Africa.; ^3^ The Guy Foundation, Dorset, UK.; ^4^ Discipline of Physics, School of Agriculture and Science, University of KwaZulu-Natal, Durban 4001, South Africa.; ^5^ National Institute for Theoretical and Computational Sciences (NITheCS), KZN Node, University of KwaZulu-Natal, Durban 4001 South Africa.; ^6^ Research Centre for Optimal Health, University of Westminster, London, UK.; ^7^ School of Data Science and Computational Thinking, Stellenbosch University, Stellenbosch 7600, South Africa.

## Abstract

All life on Earth shares an evolution that is coupled to specific environmental conditions, including the geomagnetic field, which would suggest that biological materials have evolved some degree of magnetic sensitivity. Mounting evidence supports the idea that living organisms can detect and respond to weak magnetic fields, and the radical pair mechanism provides a plausible quantum-level basis for magnetic field sensitivity in biological processes. In the context of evolution, however, fundamental questions remain. How does natural selection act on quantum phenomena such as spin dynamics? What evolutionary pressures have tuned or preserved magnetic sensitivity at the quantum level? In this paper, we offer some potential answers, by closer inspection of the spin Hamiltonian central to the radical pair mechanism. We focus on the fact that hyperfine coupling constants are determined by the local protein environment, which is specified by genetic sequence. Variations in these parameters can alter reactive oxygen species (ROS) outcomes, providing a potential pathway by which quantum-level effects influence organism fitness and possible selection. As proof of principle, we employ a simple qualitative model to demonstrate how variations in nuclear spin, hyperfine coupling strength, and anisotropy might alter ROS levels under different magnetic field conditions. Although these ideas remain speculative, they suggest a framework for investigating how magnetic environments may have influenced the evolution of biological function, with implications for health, disease, and adaptation to altered magnetic environments.

## Introduction

Biological organisms have evolved over billions of years in a very specific electromagnetic environment. Evolution is the complex interaction between biological materials and their environment; thus, biological systems must be coupled to the Earth’s rhythms and characteristics, one of which is the geomagnetic field [1]. It is becoming increasingly clear that electromagnetic processes are central to living systems, with biological function underpinned by charge transfer through molecular systems. This suggests that magnetic sensitivity may be deeply embedded in biology, reflecting evolutionary adaptation to a specific electromagnetic niche [2]. This is borne out by the growing evidence that weak magnetic fields—where by “weak” we mean roughly Earth strength and smaller (in the range micro- to nanotesla)—can have a direct effect on biological systems, potentially through the modulation of quantum spin in the materials that constitute these systems [3]. Spin is the property of quantum particles such as electrons and protons that describes their response to a magnetic field. Spin has already played a role in revolutionizing medical techniques through the invention of magnetic resonance imaging (MRI). The difference is that MRI machines exploit the spin-polarizing effects of strong magnetic fields [4]. What is still not yet properly understood is exactly how weak magnetic fields, through mechanisms such as radical pairs, are implicated in biological and physiological—and, indeed, even psychological—outcomes [3].

The experimental evidence is suggestive. A number of fundamental biological processes are influenced by weak magnetic fields. A recent review documents the effects of hypomagnetic fields on mitochondrial function, suggesting that mitochondria are a key target of altered magnetic fields [5]. Microtubules have also been shown to exhibit magnetic field effects [6,7]. Circadian rhythms are sensitive to magnetic fields [8,9]. Stem cell development, both neurogenesis and other, is modulated by weak magnetic fields; a phenomenon that is thought to be mediated by reactive oxygen species (ROS) [10–13]. Tadpole embryogenesis also appears to be influenced by hypomagnetic fields [14]. Magnetic fields have been shown to be important to a number of different biological functions with further implications. Birds can sense the geomagnetic field and potentially use it as a compass in their migrations [15–18]. Experiments show that the human brain also responds to perturbations of the geomagnetic field. Magnetic field changes result in a decrease in amplitude of alpha frequency (8 to 13 Hz) brain waves, which are related to the brain’s processing of external stimuli [19]. Research also suggests that disruptions to the Earth’s field caused by geomagnetic storms correlate with physiological and psychological changes, including increased instances of suicide (though it is unclear whether the effect is due to direct magnetic effects or increased solar radiation) [20–22]. It should be noted, however, that this correlation by no means proves a mechanistic connection.

Magnetic fields, it would appear, are implicated in shaping both physical and mental health. Interest in the biological effects of weak magnetic fields is also expanding in the theoretical context. A number of mechanisms have been proposed to account for magnetic field sensitivity in living systems. These include magnetite-based models, in which biogenic magnetic nanoparticles provide a direct means of detecting external fields [23,24], as well as hybrid frameworks that combine such effects with spin-dependent chemical processes [25]. Among these, the radical pair mechanism has emerged as one of the most prominent and widely studied, being applied to a number of diverse contexts [3,6,26–28]. The radical pair mechanism is one of the primary topics in the field of quantum biology. Definitions of quantum biology differ, but it might be said that it encompasses the study of novel quantum effects—such as superposition, coherence, tunneling, and entanglement—in the remarkable materials that constitute biological systems [29]. Quantum biology encompasses a wide range of topics including photosynthesis [30–35], enzyme catalysis [36,37], DNA mutation [38,39], receptor binding [40–42], microtubule and mitochondrial function [43–46], magnetoreception [16,47–49], regulation of the production of ROS [50,51], calcium ion storage and release [52–54], and, potentially, consciousness [55]. Compared to highly speculative proposals in quantum biology, such as quantum theories of consciousness, spin-dependent radical pair biochemistry is comparatively well supported both experimentally and theoretically, particularly in the context of magnetoreception.

There have also been some attempts to understand whether evolution played a role in tuning quantum parameters in biological systems, and if so by what mechanisms this could have been achieved. By “quantum effects in evolution” we do not imply a nonclassical theory of evolution itself, but rather that quantum-level processes within ordinary biochemistry may influence physiological traits that are subsequently acted upon by conventional evolutionary mechanisms such as natural selection. Some approaches to this question consider how quantum effects may directly influence DNA mutation [38,39,56], providing raw material for selection at the genetic level. But evolution may also have played a role in tuning certain quantum parameters in biological systems, under the influence of different selection pressures. For example, photosynthesis involves the movement of energy along electron transport chains, for the purpose of adenosine triphosphate (ATP) synthesis. Energy is transported between light-sensitive molecules known as chromophores, which are embedded in proteins. There has been a great deal of investigation into the question of whether this energy transport utilizes quantum coherence to enhance efficiency [30–35]. While there is yet to be a definitive answer to this question, these transport processes depend on the architectures of the relevant chromophores and their orientations and separation distances. It is thus conceivable that, against the selective pressure of bioenergetic management in photosynthesis, evolution may have influenced the geometry of chromophore networks to maintain quantum coherence.

Similarly, evolution may have exploited the magnetic parameters that quantify radical pair reactions, refining sensitivity to geomagnetic fields. For example, differential metabolic fractionation of isotopes in certain species has been hypothesized to affect the evolution of migration, by improving the sensitivity of the radical pair compass [57]. Recent interest in how avoided crossings in radical pair spin states can confer a functional biological advantage such as increased compass sensitivity in magnetoreception also suggests that quantum effects may have played an evolutionary role in biological organisms [58]. In this paper, we examine more closely how a terrestrial environment may have influenced the magnetic parameters of biological materials. In the “The radical pair mechanism: How is biology sensitive to weak magnetic fields?” section, we first describe the molecular machinery by which biological systems might manifest magnetic sensitivity. In particular, we focus on the radical pair and spin chemistry. In the “From spin to evolution: What are the tunable magnetic parameters in biological materials?” section, we clarify which magnetic parameters can be tuned in radical pair reactions by analyzing the different terms of the spin Hamiltonian that describes radical dynamics, and how these terms depend on the physical properties of the molecules the radicals are situated in. Evolution acts at the level of DNA rather than directly on electron spin. We therefore make explicit the link between the two: Genetic variation and mutation alter the proteins encoded by DNA, which in turn define the local electronic and nuclear environment of radical pairs. Changes in this protein environment modify hyperfine coupling in multiple ways, thereby altering the reaction pathways and products of radical-based biochemistry. Finally, in the “Sensing and signaling: What selection pressures act on quantum parameters?” section, we consider how natural selection could act on this system, proposing that mutations that generate hyperfine environments that most effectively regulate ROS in the presence of the geomagnetic field would confer a fitness advantage. Such selection pressures were likely particularly important early in evolution, when rising oxygen levels made robust ROS management critical. To conclude, we discuss the implications of this sensitivity, in terms of our understanding of health within our terrestrial environment, as well what this means for humans as a multi-planetary species.

## Terrestrial Fine-Tuning of Magnetic Parameters

### The radical pair mechanism: How is biology sensitive to weak magnetic fields?

While there is ample experimental evidence that living organisms—from plants to humans—display some sensitivity to weak magnetic fields, the exact mechanism of this sensitivity remains debatable. Different explanations have been proposed, including the important roles that iron compounds likely play, suggesting that magnetic sensitivity may manifest in more than one way across biological systems [23–25]. In this paper, however, we focus on the radical pair mechanism—first developed in the context of spin chemistry to describe how magnetic fields influence the yields of chemical reactions [59,60]—as a particularly well-developed and tractable model within quantum biology. This framework provides a useful way to explore how evolutionary processes may have exploited effects such as spin dynamics and coherence. A radical pair consists of 2 unpaired electrons that are spatially separated but spin correlated. This spin correlation can take the form of a singlet state or 1 of 3 triplet states. These states describe different alignments of the spins of each electron with respect to the other. Once the radical pair has been formed, it undergoes singlet–triplet oscillation due to the interaction of the electron spins with environmental factors such as the external magnetic field (Zeeman effect) and the magnetic moments of the surrounding nuclei in the molecule (hyperfine effect) as well as interactions between the radicals themselves (exchange and dipole interactions). In reactions involving radical pairs, the relative spin states determine what reactions can proceed. For example, 2 radicals can recombine to form a bond if their spins are arranged in a compatible (singlet) configuration. Thus, singlet and triplet states result in different chemical outcomes. Because the ratio of these spin states can be altered by the application of magnetic fields, these fields can alter the outcomes of chemical reactions [16,59,60]. The mechanism is described in some detail in Fig. 1. At its simplest, however, the radical pair mechanism depends on 2 main principles: that magnetic fields can modulate spin states, and that spin states can modulate the outcome of chemical reactions.

**Fig. 1.**
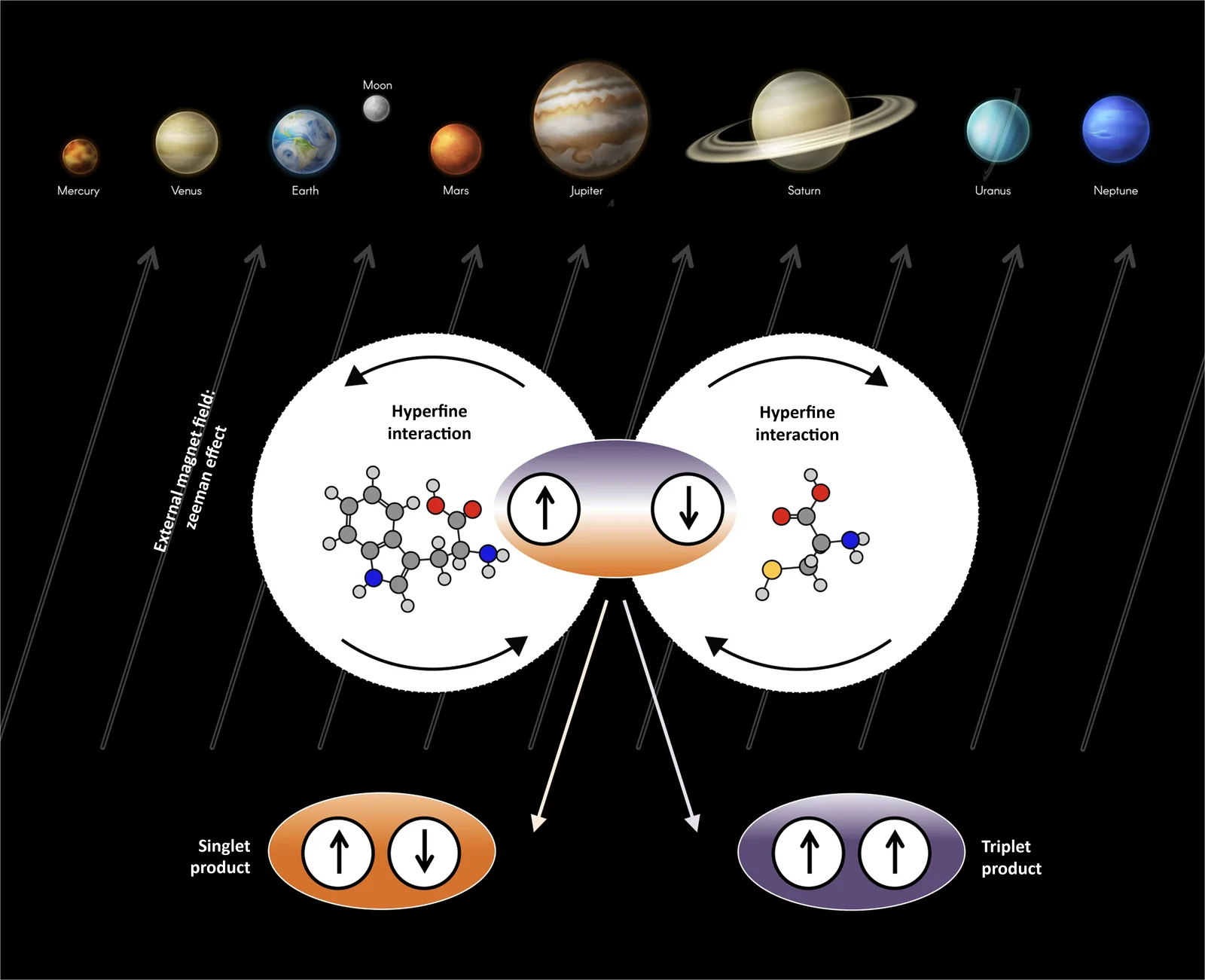
An illustration of the radical pair mechanism, with electrons of the pair given by ↑ and ↓ to represent their spin. The creation of the radical pair involves an electron transfer facilitated by either light or chemical reaction, resulting in a pair of electrons that are spatially separated but spin-correlated. This collective spin state can be either a singlet or triplet state, which differs in the alignment between the 2 spins. The initial spin state is then modulated through the interaction of the electron spins with their environment. There are a number of different interactions that can take place to drive this modulation. The radical pair mechanism, as it appears in the context of quantum biology, often prioritizes 2 of these interactions: the Zeeman and hyperfine effects. The Zeeman effect is the interaction of electron spin with a static external field, such as the geomagnetic field. The hyperfine effect is the interaction of electron spin with the magnetic moments of the surrounding nuclei in nearby amino acids, such as tryptophan or cysteine. These interactions result in the radical pair oscillating between singlet and triplet states. The final step of the radical pair mechanism is the conversion of this spin character into a measurable chemical outcome. The radical pair is an intermediate state. Chemical binding is restricted by the spin state, which means that a magnetic field can change the outcome of a chemical reaction by changing the spin state of the intermediate radical pair [16,59,60]. Thus the ratio of singlet to triplet states, determined by the Zeeman and hyperfine interactions, can alter the yields of chemical reactions. This means that changing the parameters of the external magnetic field (travel to a new planet) or the local hyperfine fields (different molecules in the radical pair vicinity) will change the ratio of singlet to triplet, in turn changing the chemical outcome and any biological function dependent on this outcome. Travel to any of the other planets in our solar system will result in some perturbation to this singlet to triplet ratio, as the magnetic fields of the different planets range from negligible to stronger than the Earth’s field.

Not long after the initial discoveries and development of spin chemistry [59,60], the radical pair mechanism was adopted as a potential mechanism to explain the avian compass [47]. The avian compass appears to be unaffected by a switch in north and south poles, which has led to the suggestion that it is an inclination compass, and location is determined by the angle of the Earth’s magnetic field lines [16]. The radical pair mechanism is currently the model that best describes this and other aspects of the avian compass, although there has been some contention that a three radical model would address some of the many issues that plague the two radical model [61,62]. In short, it is hypothesized that light incident on cryptochrome proteins in the eyes of birds causes the excitation and transfer of an electron, forming a radical pair. The alignment of the bird in the Earth’s magnetic field then modulates the ratio of singlet and triplet spin states through the Zeeman effect, which ultimately reflects in a different chemical outcome that translates into a specific biological signaling state [16,47–49]. There have been many papers published on the topic, although there remains an absence of definitive experimental verification, with the majority of experiments targeting bird behavior rather than chemical or biological markers [16,63,64]. A recent study demonstrated that flavin-based autofluorescence in cells is sensitive to weak magnetic fields, a hallmark of the radical pair mechanism [65]. The main candidate molecule for radical pair chemistry in biological systems has been cryptochrome, in which the radicals formed involve flavin adenine dinucleotide (FAD), tryptophan residues, and potentially superoxide [3,49,64,66]. While most of the focus has remained on cryptochrome, there are a number of other potential candidates for radical production involving amino acid residues such as tyrosine [3]. For an excellent review of radical pairs in biology, including potential radical candidates, see the paper by Zadeh-Haghighi and Simon [3]. The radical pair model of avian magnetoreception has been a subject of study for over 4 decades. However, in the past few years, there has been growing interest in applying the radical pair mechanism to novel biological contexts [6,26–28] such as modulation of ROS.

### From spin to evolution: What are the tunable magnetic parameters in biological materials?

Despite some remaining debate, the radical pair mechanism (or three radical alternative) is a plausible model with which to understand magnetic sensitivity in biological organisms. However, in order for this magnetic sensitivity to evolve, radical pair reactions need to be susceptible to DNA mutations that confer some evolutionary advantage. To investigate how radical pair parameters depend on their biological context, we examine the terms of the spin Hamiltonian for a standard radical pair. The Hamiltonian includes the following spin interactions,

$$H=H_{\text{Zeeman}}+H_{\text{Hyperfine}}+H_{\text{Dipolar}}+H_{\text{Exchange}}+H_{\text{Nuclear}}.$$ (1)

In our further discussion, we neglect to include $H_{\text{Nuclear}}$, the interaction of the nuclear spins with the external field, as the gyromagnetic ratio is much smaller than in the electronic case [67]. Each of the remaining terms have a number of parameters that influence magnetic sensitivity,

$$H=\sum_{i}^{2}\gamma_{e}B\cdot S_{i}+\sum_{i}^{2}\sum_{k}^{N_{k}}S_{i}\cdot A_{\mathrm{ik}}\cdot I_{\mathrm{ik}}+S_{1}\cdot D\cdot S_{2}-2J\;S_{1}\cdot S_{2}.$$ (2)

The first term represents the Zeeman interaction of the radical electron spins $S_{1}$ and $S_{2}$ with the external magnetic field B, with electron gyromagnetic ratio $\gamma_{e}=\frac{-g\mu_{B}}{\hbar }$, where g=2 and $\mu_{B}$ is the Bohr magneton. The second term gives the hyperfine interaction, where each electron interacts with $N_{k}$ surrounding nuclei and $A_{\mathrm{ik}}$ is the hyperfine coupling tensor for each interaction. $I_{\mathrm{ik}}$ denotes the spin operator of the kth nucleus coupled to electron i, where $i\in \left\{1,\;2\right\}$ labels the 2 radicals and k runs over the nuclei in the local hyperfine environment of each radical. The last 2 terms of the Hamiltonian, the dipolar and exchange terms, represent interactions between the 2 electron spins, with D the anisotropic dipolar coupling tensor and J the exchange coupling constant. With the exception of the Zeeman term, the spin interactions depend on the properties of the material in which they are located. For the hyperfine interaction, this includes the type of nuclei that the radicals interact with (their spin as well as their hyperfine coupling strength). The dipolar and exchange interaction both rely on radical separation. One way in which DNA mutation may have an effect on radical pair reactions is by altering the structure of the proteins in which these radicals are embedded. This would change the hyperfine environment as well as the radical orientation and separation.

For the purpose of this paper, we consider a simplified example. In many of the models employed in radical pair research, the dipole and exchange interaction are commonly discounted, either due to sufficient separation of the electrons or because the separation is optimal for the effects to cancel [6,7,26–28,68–70]. Although this may not necessarily be the case in reality, if the radicals are sufficiently separated, then, in the context of evolution, the primary interaction of interest is the hyperfine interaction. The hyperfine interaction reflects the local magnetic environment of the radicals and can be influenced by the particular protein in which the radical pair is formed. An example of this is cryptochrome, the protein thought to involved in the avian magnetic compass. Cryptochrome-based radical pairs involve FAD and tryptophan residues [3,49,64]. There has been some research suggesting that the magnetic sensitivity of the avian compass is sharply increased by the specific anisotropic hyperfine environment. This “magnetic needle” exploits a uniquely quantum effect known as avoided crossings, which has to do with the interconversion of specific spin states in radical pairs [58]. An avoided crossing leads to strong mixing between spin states, producing a pronounced and often narrow feature—such as a dip or spike—in the singlet yield [71–73]. In cryptochrome, the hyperfine environment includes 2 nitrogen atoms, whose anisotropic hyperfine interactions with the radical pair have been suggested to contribute to avoided level crossings, resulting in a sharp spike in magnetic sensitivity [58]. There has been some contention over the theory, due to the fact that in the initial model both the inter-radical (dipolar and exchange) interactions were taken to be negligible as well as the condition that the radical pair lifetime persists beyond a few microseconds [58]. A number of subsequent studies have attempted to address these issues [74–78]. While still a subject of debate, if avoided crossings do play a role in optimizing features of the radical pair, it would be expected that swapping out specific nuclei with appropriate anisotropic contributions would have an effect. Recent experimental research into the magnetic sensitivity of different variations of cryptochromes from migratory and nonmigratory species showed that most mutations in specific sites had little effect. Interestingly, however, mutation of a tryptophan to a phenylalanine showed a marked increase in the protein’s magnetic field sensitivity [79].

The protein-specific hyperfine interaction thus offers various parameters by which to modulate radical pair spin dynamics. In Figs. 2 to 4, we consider 3 different parameters: (a) the spin of the interacting nuclei, (b) the hyperfine coupling strength, and (c) the hyperfine anisotropy. Using a simple toy model for the radical pair mechanism, we illustrate the influence of each of these parameters. To investigate radical pair dynamics, we consider singlet product as our outcome of interest, rather than directional sensitivity. We do this because we are interested in biological contexts that include signaling more broadly rather than merely magnetoreception. For singlet product yield, we follow the example of Tiersch and Briegel [67]

$$\Phi_{S}=\int_{0}^{\infty }{\text{dtke}}^{-\mathrm{kt}}\langle S|\rho_{e}\left(t\right)|S\rangle ,$$ (3)

where $|S\rangle$ is the singlet state, $P^{S}=|S\rangle \langle S|$ is the corresponding singlet projection operator, $\rho_{e}\left(t\right)$ is the reduced density matrix of the electrons, and $\frac{1}{k}$ is the radical pair lifetime. All simulations in this study were performed using the QuTiP 5 (Quantum Toolbox in Python) framework [80]. We summarize here the parameters used in the toy model simulations shown in Figs. 2 to 6. The radical pair was initialized in the singlet state, with the radical pair lifetime taken to be $\frac{1}{k}$ = 10 μm. The external magnetic field was varied over the ranges shown in the figures. The hyperfine couplings used in each figure were as follows: In Figs. 2 and 5, the isotropic coupling was set at A=0.5 mT; in Figs. 3, 4, and 6, details are included in the graphs.

**Fig. 2.**
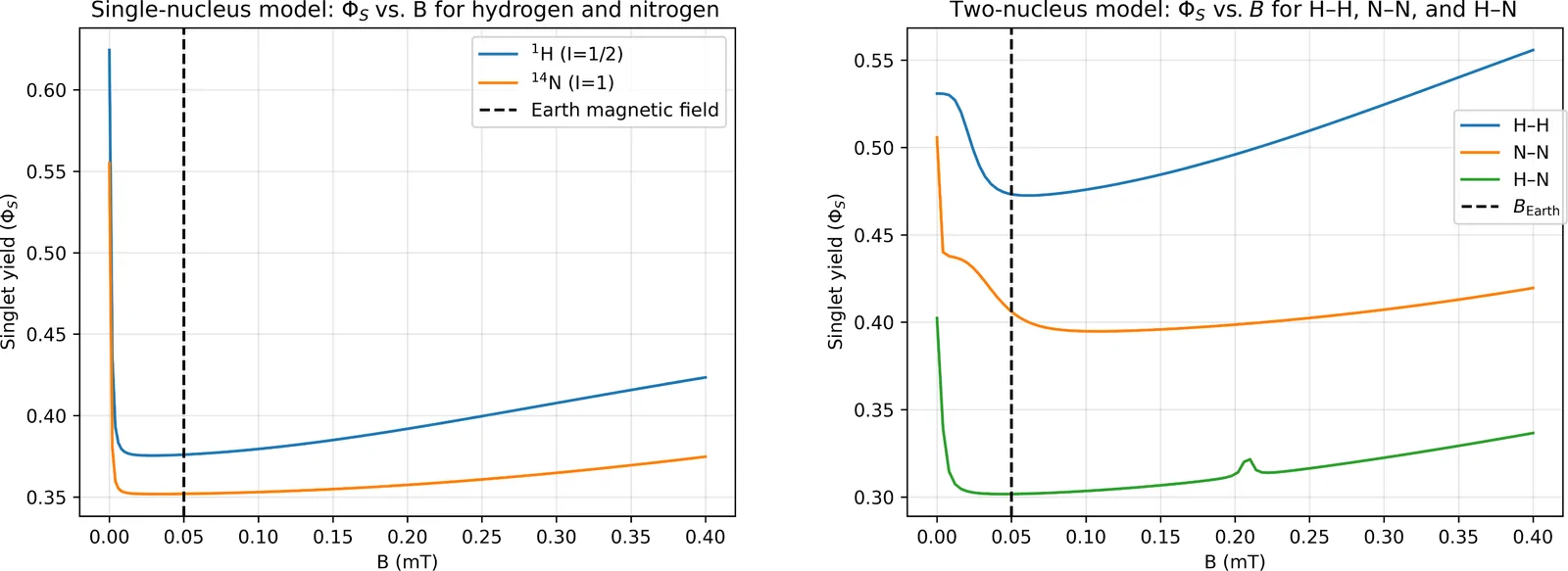
Singlet yield as a function of magnetic field strength for nuclei with spin $\frac{1}{2}$ (e.g., ^1^H) and spin 1 (e.g., ^14^N). Although nuclear spin and hyperfine coupling strength are related, here, spin is considered independently to isolate its effect on radical dynamics. Spin-$\frac{1}{2}$ nuclei generally produce higher singlet yields than spin-1 nuclei, an effect observed for both single-nucleus and 2-nucleus models and amplified with additional nuclei. On the other hand, radical pairs with nuclei of different spin on either radical (H–N) show the lowest levels of singlet yield.

**Fig. 3.**
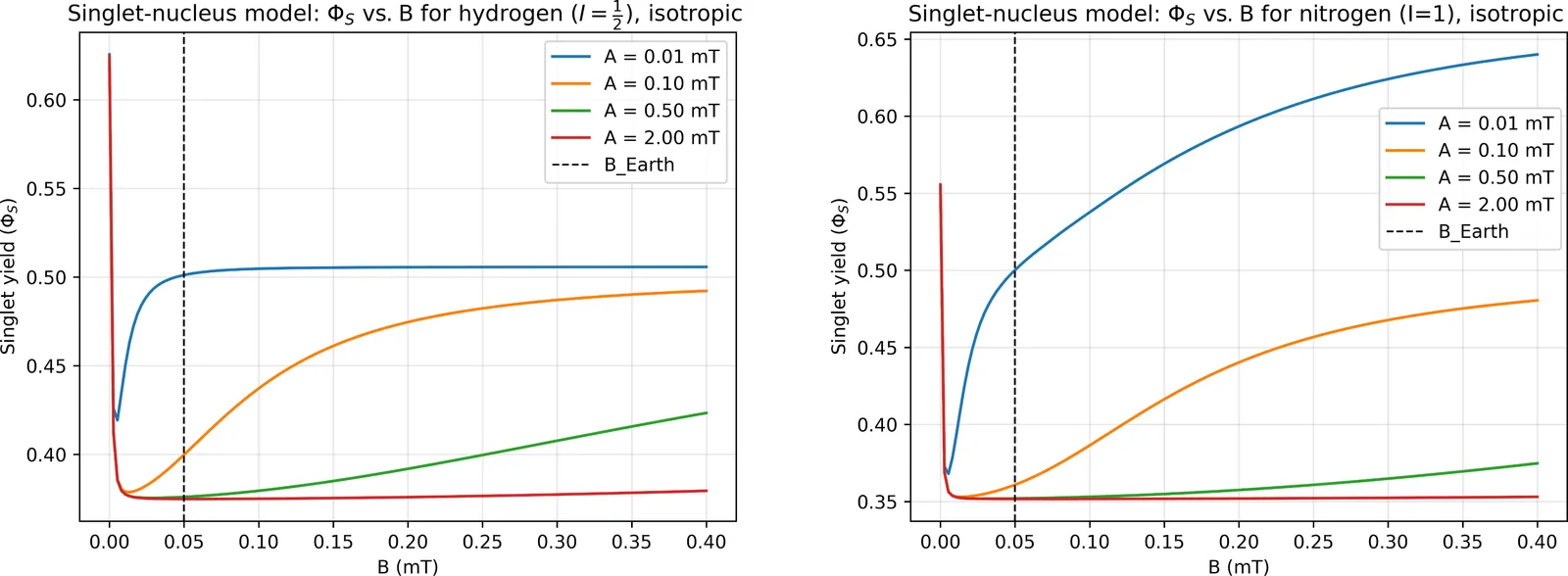
Singlet yield as a function of magnetic field strength for varying isotropic hyperfine coupling strengths, shown for nuclei with spin $\frac{1}{2}$ and spin 1. For small hyperfine coupling constants, the singlet yield reaches a minimum before increasing sharply as the field approaches Earth strength (vertical dotted line), with weaker couplings producing the highest overall yields. As coupling strength increases, the minimum singlet yield becomes more pronounced and the subsequent rise around Earth-strength fields becomes more gradual. Very large coupling strengths (red line) exhibit a sustained singlet minimum across the full external field range. In this toy model, singlet yield—particularly for small coupling constants—also varies across the Earth’s geomagnetic field range (0.025 to 0.065 mT) [13]. This may motivate future investigation of whether magnetic field-dependent spin chemistry contributes, alongside many overlapping environmental and biochemical factors, to latitude-dependent biological—including disease responses—variation. While spin-$\frac{1}{2}$ nuclei generally yield higher singlet fractions than spin-1 nuclei, consistent with Fig. 2, this trend reverses at the weakest coupling strength: The spin-$\frac{1}{2}$ yield plateaus beyond Earth-strength fields, whereas the spin-1 yield continues to increase at higher fields. We have based our range of hyperfine coupling constants on the information for organic molecules in the paper by Hiscock et al. [58].

**Fig. 4.**
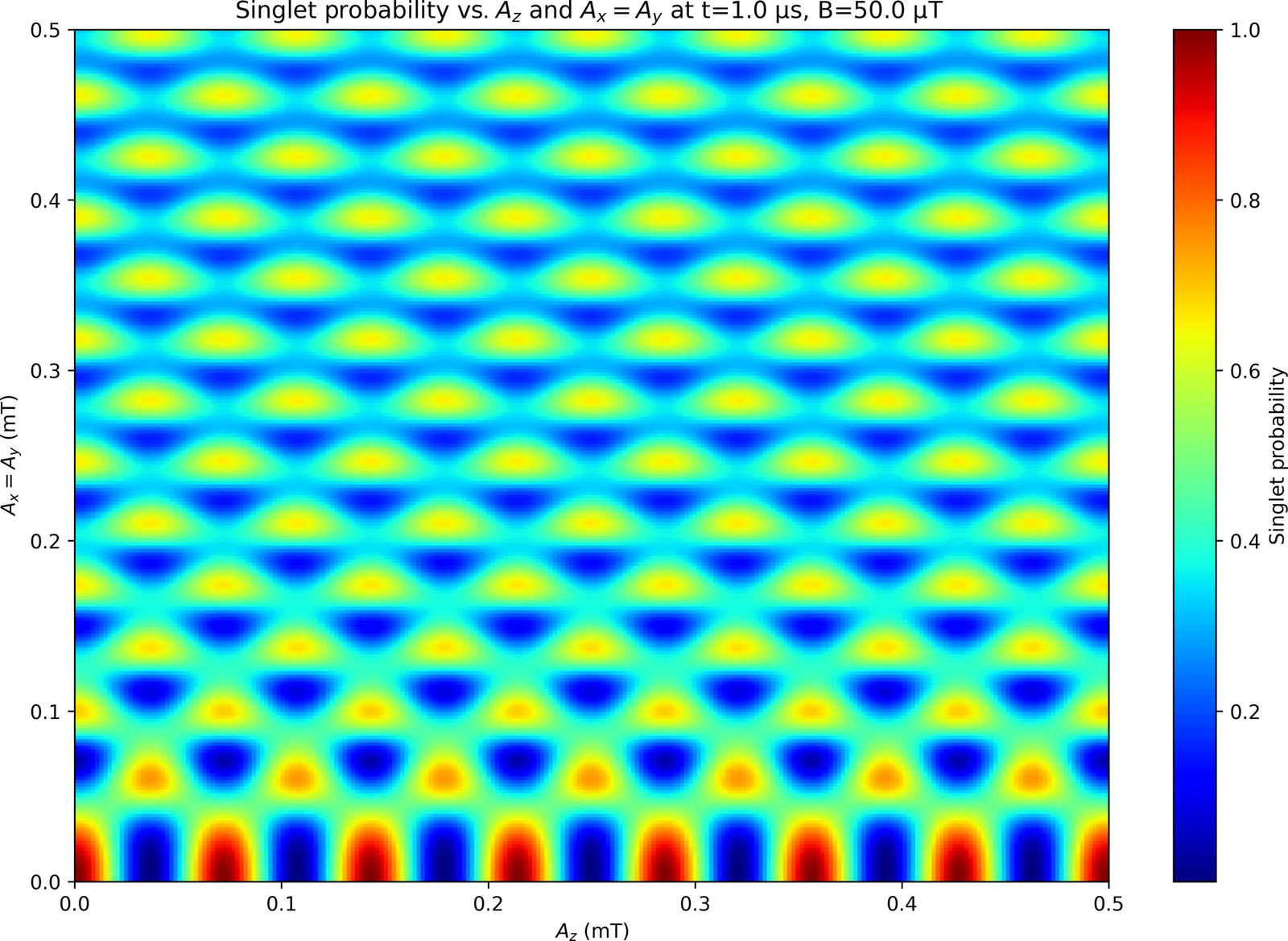
Effect of hyperfine anisotropy on singlet–triplet ratios. The singlet probability is shown for different combinations of hyperfine tensor components, illustrating how anisotropy fine-tunes radical pair outcomes. Hyperfine anisotropy has been proposed to enhance magnetic sensitivity, potentially generating sharp magnetic responses via avoided crossings between spin states [58]. Here, varying $A_{x}$, $A_{y}$, and $A_{z}$ demonstrates that different tensor components have distinct effects on singlet probability: The singlet yield can be either maximized or minimized. The precise structure of the hyperfine tensor is therefore critical in determining singlet probability under a given external magnetic field. It should be noted that these results represent single points in time.

**Fig. 5.**
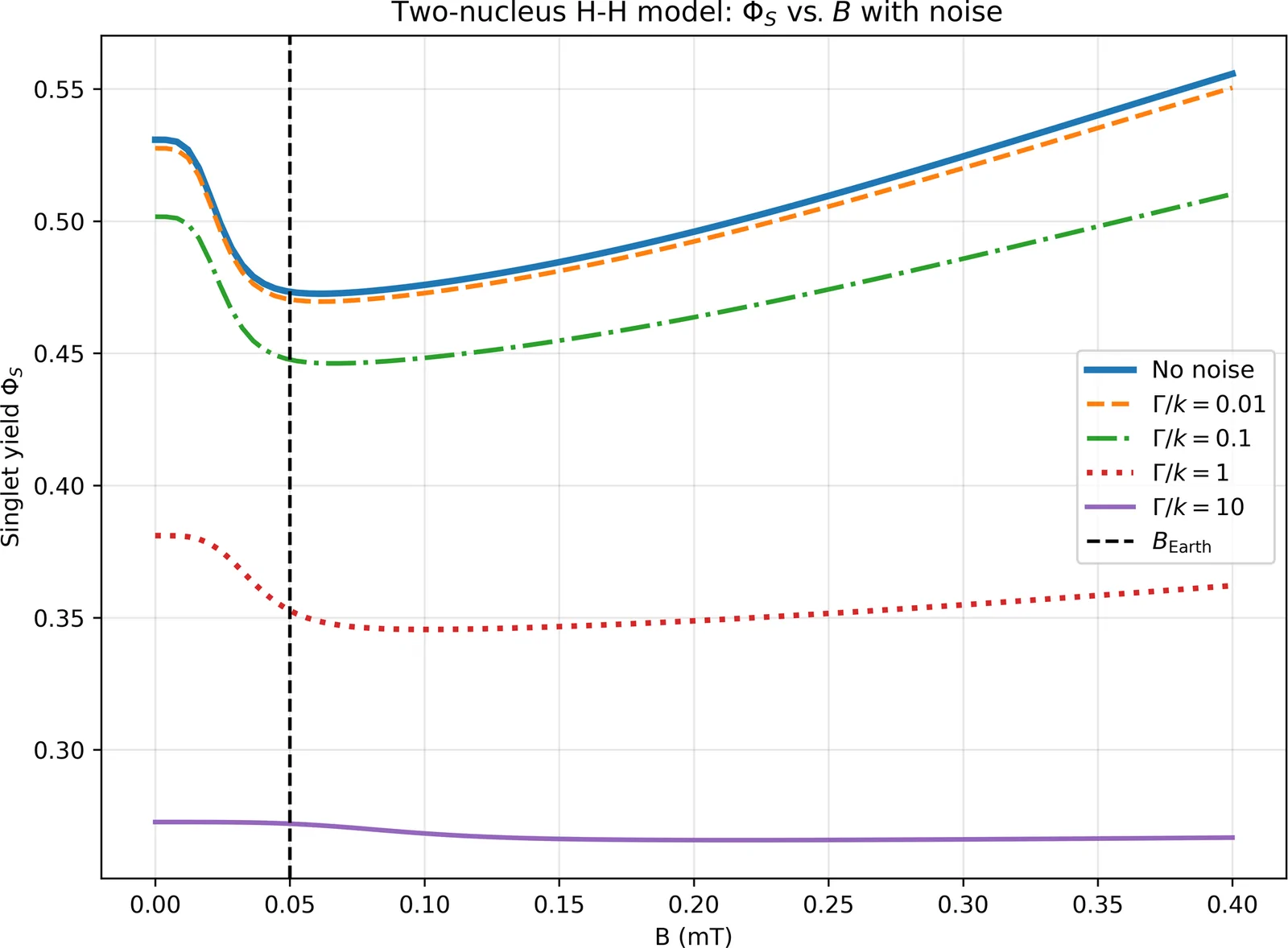
Effect of phenomenological noise on the singlet yield in the 2-nucleus H–H radical pair model. The singlet yield is shown as a function of magnetic field strength B for the noiseless case and for a generic local Lindblad noise model acting on both radical electrons, with Γ/k= 0.01, 0.1, 1, and 10. Here, k is the recombination rate and Γ is the decoherence rate. The regimes Γ/k≪1, Γ/k∼1, and Γ/k≫1 correspond respectively to weak decoherence, intermediate decoherence competing with recombination dynamics, and strong decoherence that rapidly suppresses spin coherence before recombination occurs. The dip structure near the Earth-field scale remains visible for weak noise but is progressively broadened and suppressed as Γ approaches and exceeds *k*.

**Fig. 6.**
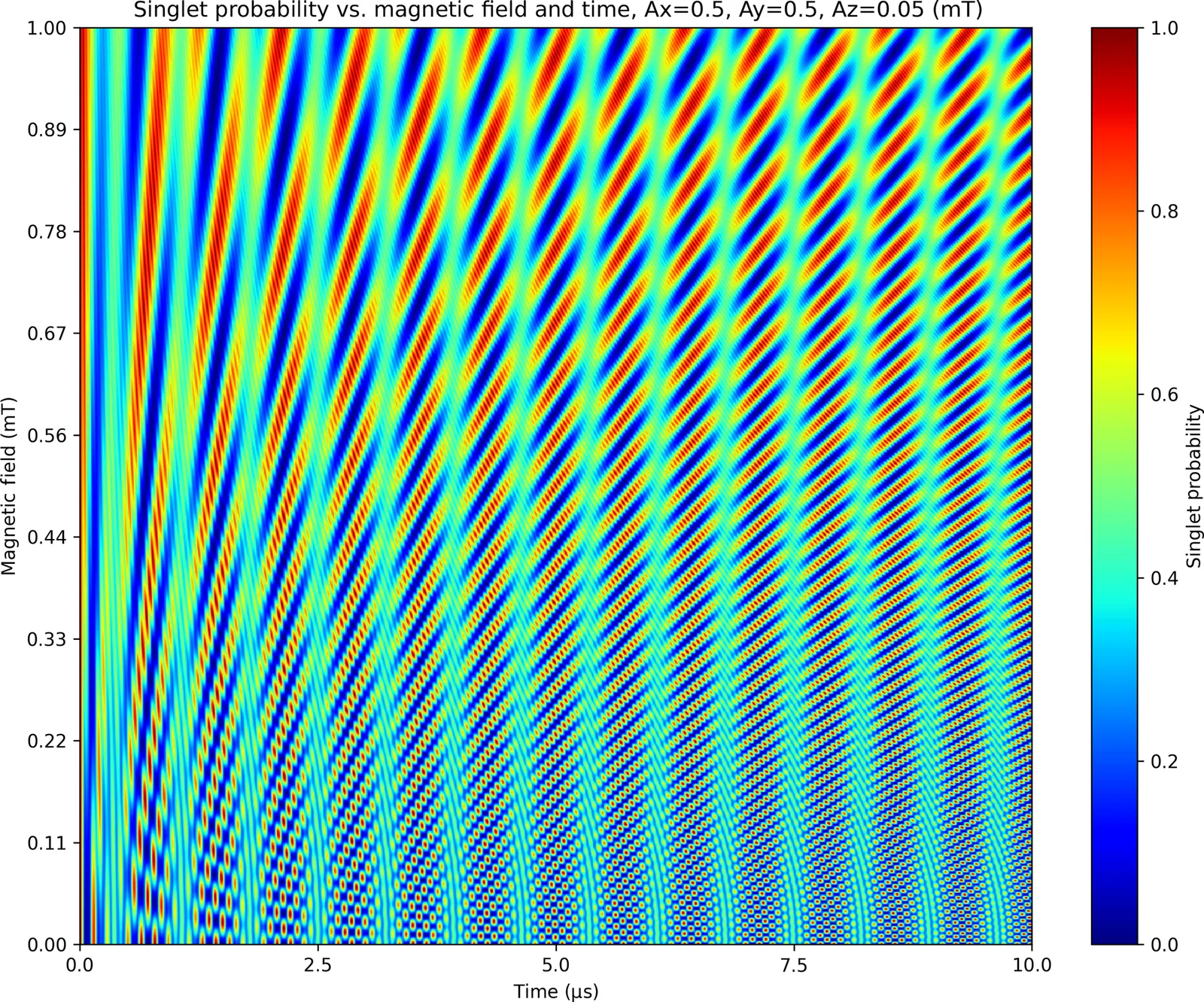
Time evolution of singlet probability over 10 μs under varying external magnetic fields. The graph illustrates how singlet probability evolves across a range of field strengths. At different field magnitudes, the singlet yield—and thus the associated ROS balance—can vary nonmonotonically with increasing field strength, consistent with experimental observations that different fields produce opposite effects on ROS yields and subsequent stem cell proliferation and differentiation [13]. While the present model employs approximate hyperfine couplings, it is intended to demonstrate the principle of field-dependent oscillation rather than exact quantitative predictions.

The hyperfine interaction will differ depending on the spin of the nuclei interacting with each of the radicals, for example, common biological elements such as hydrogen and nitrogen-14 have nuclear spin of $\frac{1}{2}$ and 1, respectively. The differential effect of nuclear spin on radical dynamics has been the basis of a number of studies highlighting interesting isotope effects in biological systems. In Fig. 2, we illustrate this simply by comparing the singlet yield for nuclei with spin $\frac{1}{2}$ and spin 1, across a range of magnetic field strengths. In this toy model and parameter regime, nuclei with spin $\frac{1}{2}$, such as hydrogen, have a higher singlet yield than spin-1 nuclei such as nitrogen. This persists for a model with a single nucleus on one of the radicals, as well as a model with 2 nuclei, one each on a radical, with the effect amplified by adding nuclei. On the other hand, radical pairs with nuclei of different spin on either radical—one hydrogen and one nitrogen for example—show the lowest levels of singlet yield.

We then consider the case of coupling strength and illustrate this with a simple example in Fig. 3, where we consider isotropic hyperfine coupling of different strengths, again for nuclei with spin of 1 and $\frac{1}{2}$. For smaller hyperfine coupling constants, the singlet yield reaches a minimum before increasing sharply as the field strength approaches Earth strength. A plausible explanation for this minimum is the competition between hyperfine-driven singlet–triplet mixing and Zeeman splitting by the external field. In the weak-field regime, where the Zeeman interaction is comparable to the hyperfine scale, singlet–triplet interconversion can be enhanced, reducing the singlet product yield. As the field strength increases further, Zeeman splitting increasingly separates the spin states and suppresses some of the mixing pathways, leading to recovery in the singlet yield. In this sense, the minimum reflects a crossover between internal and external magnetic interactions rather than a unique resonance in the strict sense. There is also some indication that, in this toy model, singlet yield varies across the Earth’s geomagnetic range (0.025 to 0.065 mT). We note, however, that any possible connection to latitude-dependent disease patterns remains speculative and would require much more detailed biological modeling and experimental validation. There are some similarities between the spin 1 and $\frac{1}{2}$ cases, especially for larger coupling constants, with spin-$\frac{1}{2}$ giving greater singlet yields, as in Fig. 2. However, it interesting to note that this does not hold for the weakest coupling constant. In this case, while singlet yield is indeed greater for the spin-$\frac{1}{2}$ case up until Earth-strength fields, this rapidly flattens out, whereas the spin-1 singlet yield continues to increase at larger fields. Singlet–triplet mixing in biological materials that evolved on Earth reflect the interplay between the Earth’s field and the magnetic properties of these materials. This does not mean that other versions of biology would not have evolved in a different magnetic field, but only that the magnetic parameters of biological materials as we know them may have been optimized in relation to this specific field.

Finally, we investigate how the hyperfine anisotropy has an effect on singlet–triplet ratios. As previously mentioned, the hyperfine anisotropy can play a big role in fine-tuning outcomes of the radical pair model. For example, it has been suggested that the comparatively large $A_{Z}$ component contributed by a seminal nitrogen in cryptochrome proteins creates a sharp spike in magnetic sensitivity, ostensibly facilitated by avoided crossings between spin states [58]. In Fig. 4, we illustrate this more generally to show why certain anisotropic combinations may have been selected for in the geomagnetic field. It is clear that the different components of the hyperfine tensor have very different effects on singlet probability. Indeed, singlet state can be maximized or minimized for specific values of $A_{z}$ in relation to $A_{x}$ and $A_{y}$. The specific form of the hyperfine tensor is instrumental in determining the singlet probability for a given external magnetic field.

The calculations presented thus far are based on a deliberately minimal radical pair model intended to illustrate how hyperfine parameters can shape spin-selective reaction outcomes and thus act as a way for evolution to act on quantum level properties. To examine the robustness of our coherent toy model features against environmental effects, we now introduce a phenomenological local Markovian noise model acting on both radical electrons. The dynamics are described by a Lindblad master equation [81]

$$\overset{\cdot }{\rho }=-i\left[H,\;\rho \right]+\sum_{j}\left(L_{j}\rho L_{j}^{†}-\frac{1}{2}\left\{L_{j}^{†}L_{j},\;\rho \right\}\right),$$ (4)

with collapse operators

$$L_{j}\in \left\{\sqrt{\Gamma }\;S_{1_{x}},\;\sqrt{\Gamma }\;S_{1_{y}},\;\sqrt{\Gamma }\;S_{1_{z}},\;\sqrt{\Gamma }\;S_{2_{x}},\;\sqrt{\Gamma }\;S_{2_{y}},\;\sqrt{\Gamma }\;S_{2_{z}}\right\}.$$ (5)

Here, Γ is an effective electron decoherence rate and $S_{1\alpha },S_{2\alpha }$ are the spin-$\frac{1}{2}$ operators of the first and second electron in the radical pair. This local noise model provides a simple phenomenological description of combined dephasing- and relaxation-like processes. In contrast to a purely coherent model, the addition of noise allows us to examine how the singlet yield changes when the decoherence timescale becomes comparable to the radical pair lifetime. As shown in Fig. 5 for the 2-nucleus H–H case, the trend in singlet yield (with a dip around Earth strength field) remains largely intact for weak noise (Γ<<k) but is strongly suppressed once Γ becomes comparable to or larger than the recombination rate k. The results reported in the noiseless model should thus be understood as illustrative of the case in which decoherence rates do not dominate over radical pair lifetimes. In biological settings, important environmental conditions—such as thermal fluctuations—would be expected to modify the quantitative behavior of radical pairs and may reduce the robustness of such magnetic signatures.

In our phenomenological noise model, the decoherence rate Γ is treated as an effective parameter that may itself depend on temperature and on the local molecular environment. We do not attempt to derive Γ(T) microscopically here. Temperature is likely to affect radical pair dynamics through several channels, including dephasing and relaxation rates. We emphasize that our simple noise model does not demonstrate whether coherence times sufficient for biologically meaningful radical pair effects can persist under realistic intracellular thermal and environmental noise conditions. A fuller treatment would require either an explicit bath model or a phenomenological open-system description with temperature-dependent rates. Such extensions lie beyond the scope of the present perspective but constitute an important direction for future work. In the context of evolution, it is less clear how decoherence in radical pair dynamics might have been mitigated, whether through selection for protein environments that reduce noise or—more speculatively—act in resonance to enhance singlet–triplet mixing. This uncertainty does not necessarily undermine our hypothesis of hyperfine-driven evolution, which is built on the assumption that sufficient singlet–triplet mixing persists despite decoherence effects. Rather, it reflects a broader and ongoing debate about the functional relevance of radical pairs in biochemistry.

### Sensing and signaling: What selection pressures act on quantum parameters?

In the previous section, we illustrated—with a very simple model—the magnetic parameters of the hyperfine interaction that can be tuned toward a specific spin outcome. These parameters include the nuclear spin, the hyperfine coupling strength, and the hyperfine anisotropy. All 3 of these depend on the specific proteins in which these radical pair reactions are situated, and as such can be shaped by evolution. In this section, we discuss some of the possible selection pressures that may have contributed to this evolution. Enhanced navigation and migration abilities may have supplied a possible selection pressure, tuning nuclear spin to optimize the avian compass [57]. As previously mentioned, selection for specific anisotropies in radical pair reactions located in cryptochrome proteins may also have enhanced the magnetic sensitivity of the avian compass. Hiscock et al. [58] discuss a sharp spike in compass sensitivity that depends on the anisotropy of the hyperfine interaction, specifically the large $A_{z}$ component and smaller $A_{x}$ and $A_{y}$ components. They attribute this to avoided crossings, which result from the small perturbation of the $A_{x}$ and $A_{y}$ components that couple the diagonal states of the spin Hamiltonian; for details, see the supplementary material for the paper by Hiscock et al. [58].

While the optimization of the avian compass has drawn most of the attention, we suggest that this might be a more general principle, and that evolution may have tuned the magnetic parameters of biological materials around the Earth’s magnetic field by selecting for hyperfine environments that exploit quantum phenomena such as avoided crossings for optimal biological function. In contrast to the niche selection pressure of magnetoreception, we suggest a broader selection pressure relating to signaling and inflammation mechanisms in biological materials, that is, the balance of ROS. Interestingly, it has been suggested that daily fluctuations in oxidative stress may have acted as a marked selection pressure for the evolution of the circadian clock [82], one of the key elements of which is cryptochrome, a magnetically sensitive protein. It should be noted that, while we focus on ROS in this paper, there are other relevant radical-based signaling molecules such as reactive nitrogen species (RNS) [83]. In addition to this, prior to the increase of oxygen on Earth, metals such as iron would have facilitated electron transfer reactions and potentially mediated magnetic sensitivity [84,85]. Indeed, there is some research that radical dynamics may involve metal ions, with an ion-radical mechanism being suggested as the basis for the magnetic field sensitivity of phosphorylation processes [86]. Given the fundamental importance of phosphorylation in biological systems, this could equally have served as a mechanism through which natural selection optimized radical pair parameters. Our choice of ROS balance as a selection pressure only reflects emerging interest in the subject in the context of the radical pair mechanism and quantum biology.

It is perhaps surprising that it has taken so long for interest in the radical pair mechanism to expand into new contexts, particularly in the context of medicine, given the central role of redox chemistry in living systems. Free radicals, for instance, are ubiquitous in physiological and pharmacological contexts [87]. Although they are often associated with damaging, inflammatory effects, these highly reactive species—frequently involving oxygen—also serve as essential signaling molecules within a tightly regulated homeostatic network [88]. Importantly, there is substantial experimental evidence that ROS are sensitive to a range of magnetic field conditions [89]. Only more recently has a growing body of literature sought to interpret these effects through the lens of quantum biology, in particular via the radical pair mechanism. There has been some speculation that this mechanism may involve oxygen-based radicals [64,66]. ROS are inextricably linked to metabolic processes, with the mitochondrial electron transport chain representing a primary source [51,88]. Indeed, a recent study investigating magnetic effects in recombinant human electron transfer flavoenzyme reoxidation shows that static magnetic fields modulate ROS partitioning, with changes to hydrogen peroxide and superoxide production. The authors of the study suggest that this points to a flavoprotein-based magnetic sensor that is linked to mitochondrial bioenergetics and cell physiology [50,51].

The radical pair mechanism has now been invoked across a range of emerging biological contexts, including the mechanism of lithium action in bipolar disorder, anesthesia, microtubule reorganization, and circadian rhythms [3,6,26–28]. It has also been proposed as an explanation for magnetic field effects on ROS-dependent processes such as adult neurogenesis and cognition in mice [70], where hypomagnetic field conditions are experimentally achieved through attenuation of the geomagnetic field [10,11]. The use of a two radical model, however, has elicited some criticism for the fact that the radical in question would be created in a triplet rather than a singlet state. In addition to this, lifetimes of the oxygen radical in question are very small, which throw some doubt on the efficacy of the mechanism [90]. A radical triad would mitigate some of these effects and is suggested to play a role in hypomagnetic field effects on neurogenesis [90]. What is interesting is that, in addition to neurogenesis, a number of the studies investigate the effects of weak magnetic fields on stem cells in other contexts. One study reviewed evidence for a link between non-ionizing radiation and apoptosis-induced cell proliferation [12]. Another investigated whether static weak magnetic fields might change stem cell proliferation and differentiation through modulation of ROS and heat shock proteins. The results demonstrated that different field strengths increased or decreased tissue formation in vivo [13] and furthermore that this specifically involved modulation of superoxide species [91]. Superoxide has also been shown to play a pivotal role in mammalian magnetic field signal transduction relating to circadian rhythms [92].

Origins-of-life research has also linked eukaryogenesis to rising oxygen levels on early Earth, with evidence suggesting that archaeal ancestors of eukaryotes possessed metabolic traits enabling interactions with oxygen or its derivatives. The emergence of eukaryotes—particularly through mitochondrial acquisition—likely coincided with increasing oxygen availability, enabling efficient energy metabolism while driving the evolution of redox regulation [93]. In this context, the crucial role that ROS play in signaling and inflammation suggests that ROS balance may have been an important factor in evolutionary fitness [94]. In the context of the radical pair mechanism, this means that the nuclear environments surrounding relevant radical pairs may have been selected for to optimize singlet or triplet yields—and thus different reactive species—in a given external field. The materials out of which living organisms have constructed themselves are thus structurally coupled to the field they evolved in, and the introduction of a different external field will disrupt this optimization. Consider recent experimental results that demonstrate the effects of weak magnetic fields on stem cell proliferation and differentiation via modulation of radical spin and ROS balance. The results demonstrated that different field strengths increased or decreased tissue formation in vivo [13]. Specifically, 0.2-mT fields showed decreased blastema formation in comparison to increased blastema formation in 0.5-mT fields. Figure 6 makes it clear that, at least for specific anisotropic hyperfine strengths, spin state (and thus corresponding ROS balance) oscillates over increasing field strengths. This would be consistent with the experimental finding that different fields have opposite effects on ROS yields. It should be noted that we are merely interested in the principle of this oscillation but do not assume that our graphs represent exact singlet probabilities at specific external fields, given our toy model and approximation of the hyperfine coupling. Our aim is only to illustrate how materials such as amino acids, with nuclear environments that favor a certain ROS profile with specific biological outcome (signaling, stem cell moderation), might have been more likely to be selected for.

While we have outlined a mechanism by which heritable quantum parameters might affect evolutionary fitness, there remains a lack of rigorous evolutionary analysis and experimental verification. A detailed treatment of evolutionary theory in this context is, however, inherently difficult because the proposed selection pressure—ROS balance—is both ancient and relevant across multiple biological processes and environmental inputs, making it hard to isolate its specific evolutionary trajectory. These challenges are compounded by the likelihood that key ROS-related genes were shaped during early events such as rising oxygen levels, embedding long-standing constraints that persist today [93]. At the same time, uncertainty around the relevant radical pair systems—beyond well-studied cases like cryptochrome—limits the ability to connect quantum-level parameters to specific genetic and population-level dynamics, making a comprehensive evolutionary analysis premature.

These constraints make experimental verification difficult as well. Our primary assertion—that genetic variations affecting radical pair dynamics will change ROS outcomes—could be tested by changing protein environments in a manner that changes the hyperfine environment—either by isotope substitution or by amino acid substitution—and observing the resultant ROS levels. However, candidate radical pairs in quantum biology have not been well developed, so it is difficult to perturb the interaction between hyperfine environments and applied magnetic fields if we are not clear what proteins constitute the environment. There have been some attempts to look at how selective cryptochrome mutations might influence fitness—in this case the sensitivity of the avian compass—by altering radical chemistry, with mixed results [79]. Cryptochrome proteins also show variability between migratory and nonmigratory species, which contributes to magnetic sensitivity [95]. However, one problem with identifying, for example, systematic latitude-dependent genetic variations shaped by ROS balance is that key ROS-related genes may have been shaped very early in the evolution of life, making them difficult to map to exact latitudes.

## Implications

It is already acknowledged that the geomagnetic field plays a role in protecting and facilitating life on Earth by deflecting harmful cosmic rays, among other things [1]. However, despite growing recent interest, the direct biochemical effects of weak magnetic fields have historically been underappreciated, and their mechanisms of action—whether via spin chemistry, magnetic particles, or hybrid processes—remain incompletely developed both theoretically and experimentally for contexts outside of magnetoreception. In the case of spin biochemistry, there is also a lack of well-defined radical pair candidates, and no clear explanation of how evolution may have shaped the parameters of the radical pair mechanism. If terrestrial biology has evolved to be coupled to the specific strength of Earth’s magnetic field, then the effects of weak magnetic fields on integral biological processes such as ROS signaling have significance for a number of aspects of space exploration, particularly for the case of longer-term settlement on other planets. In this instance, the new planet’s magnetic field will have an effect on physiological health but also on the health of the food that must necessarily be grown for consumption. While there is some research into the role that microgravity may play in the successful propagation of plants, there is less attention paid to the role that differing or nonexistent magnetic fields may play. Plant growth and robustness have been shown to respond to magnetic fields [96,97], a fact that will have to be considered by future populations on new planets. Magnetic fields have also been implicated in germination processes in plants [98]. That magnetic fields are implicated in processes related to reproduction would also have profound consequences for the long-term viability of human settlements in space. Magnetic fields play some role in embryogenesis, the full import of which remains to be revealed [14,99,100]. The complexities of magnetic field interactions in biological organisms will likely involve more complex solutions than the simple introduction of artificial magnetic fields to space craft, for instance.

Further research may also reveal ways in which biological systems have adapted to fluctuations in the magnetic field, evolving endogenous magnetic fields as protective mechanisms. An example of this is investigated in a paper that describes how the fast-relaxing spin of iron in photosynthetic networks can generate a magnetic field [101]. This field in turn modulates singlet–triplet conversion in radical pairs and minimizes the conversion of oxygen radicals into potentially harmful products [101]. Iron is ubiquitous in biological systems. Iron is also essential to germination processes, with iron deficiency promoting seed dormancy [102]. Iron-containing proteins such as ferritin also play a role in the management of ROS [103]. Ferritin levels are also of importance in human embryogenesis, with low birth weight correlating with low levels of ferritin [104]. In the context of embryogenesis and magnetic fields, the arrangement of iron-rich ferritin in the placenta is of particular interest, with one paper demonstrating that the magnetic susceptibility of ferritin iron increases dramatically when arranged within the placenta, as opposed to crude or purified ferritin extracts [105]. Given the effects of magnetic fields on biological systems, in particular the development of stem cells, might the arrangement of iron in the placenta have evolved to mitigate any magnetic field disruption that might harm the developing embryo? It has been suggested, for example, that endogenous iron-containing structures, such as magnetite particles, may modify the Earth’s magnetic field within biological systems. In hybrid models of magnetoreception, these particles might amplify the field experienced by nearby radical pair reactions, increasing their magnetic sensitivity [25]. There is growing interest in the importance of endogenous electric fields in generative and regenerative processes [106]. It would potentially be interesting to investigate the capacity for biological systems to produce and manage their own endogenous magnetic fields—in tandem with the geomagnetic field—especially given the role they play in modulating ROS.

A better understanding of weak magnetic fields in the biological context would benefit not only the health of future settlements on other planets but also terrestrial health. Indeed, there is increasing interest in the therapeutic potential of magnetic fields [92,107,108]. On an even more fundamental level, the Earth’s magnetic field is not completely uniform. It fluctuates over time, on short and long timescales. Geomagnetic storms, for instance, have been suggested to correlate with both physiological and psychological disruptions, although far more rigorous investigation is needed [20–22]. The reversal of the magnetic poles, which leads to an extended period of decreased magnetic field strength, has been suggested to coincide with mass extinctions, although this remains a hypothesis [1]. In addition to this, the geomagnetic field varies in magnitude at different latitudes, ranging from roughly 0.025 to 0.065 mT. If magnetic fields do play a role in the maintenance of health, it would follow that different latitudes might experience different health challenges. Given the potential role of ROS in inflammation, it might be expected that inflammatory conditions demonstrate latitude dependence. This is indeed the case, with certain biomarkers of conditions such as idiopathic inflammatory myopathies demonstrating latitude dependence, although it is not clear whether this is due to differences in ultraviolet (UV) radiation [109]. Inflammatory bowel disease also shows increased hospitalization at higher latitudes in a manner that is not fully explained by differences in season or exposure to UV light [110]. The latitudinal gradient effect is also described for multiple sclerosis, with elements of the disease being strongly positively correlated with increasing latitude toward the poles, on both sides of equator [111,112]. An analysis of infantile spasms also discovered a geographical difference in incidence, which is consistent with a latitudinal contribution to epileptogenesis, although the study was limited by a lack of representation from the Southern hemisphere [113]. Even COVID-19 displays a latitude bias, although, as with many of the diseases listed here, the hypothesis is that this is related to levels of light exposure [114]. While the correlation of inflammation with latitude is interesting in the context of magnetic field effects, we emphasize that this should be viewed with caution due to the many confounding factors, such as light exposure and weather, that also differ with latitude. We only suggest that magnetic field variations might exacerbate inflammation, and as such deserve closer scrutiny. It is difficult to overstate the importance of ROS in biological systems, both in terms of signaling and their roles in inflammation and cellular damage. However, we acknowledge that any influence of radical pair dynamics on ROS represents only one of many pathways through which ROS are regulated, and should therefore be considered as a potential addition to, rather than a replacement for, established biochemical processes. Nonetheless, a better understanding of how magnetic fields might modulate ROS could contribute to our understanding of human health and disease.

In this section, we have outlined several contexts in which magnetic fields may become relevant, from the altered magnetic environments encountered in space to terrestrial variation across latitudes. We emphasize that this discussion is necessarily speculative. Our aim is not to make definitive claims, but to highlight that if spin-dependent chemistry contributes meaningfully to biological function, then magnetic fields should be considered as a direct environmental factor influencing human health, rather than only indirectly through, for example, their role in shielding the Earth from radiation. At present, magnetic fields are—to some extent—an under-appreciated component of the environment that warrants inclusion alongside other established factors—such as the lack of gravitational fields in space or latitude-dependent light exposure—in shaping physiological function.

### Model limitations and conclusions

The simplified radical pair models presented in this paper are intended primarily as proof-of-principle demonstrations of how quantum-level magnetic parameters could, in principle, be linked to biological function and evolutionary selection. The models directly demonstrate only that varying parameters such as nuclear spin, hyperfine coupling strength, and hyperfine anisotropy can alter singlet–triplet dynamics and corresponding reaction yields under different magnetic field conditions. In this sense, the calculations establish the physical plausibility that genetically encoded changes to proteins could modulate radical pair outcomes through changes in the local hyperfine environment. A second level of caution concerns biological plausibility. There is now substantial experimental evidence that weak magnetic fields can influence ROS production, stem cell behavior, circadian rhythms, and other physiological processes. It is therefore feasible that radical pair dynamics may contribute to some of these effects. Likewise, because hyperfine interactions depend on the surrounding molecular structure, it is plausible that genetic mutations altering protein environments affect spin dynamics and associated biochemical outcomes. The idea that ROS balance may constitute a meaningful selection pressure is also consistent with the central role of redox regulation in metabolism, signaling, inflammation, and adaptation to rising oxygen levels during evolution. However, many of the broader implications discussed here remain speculative. We do not demonstrate that specific hyperfine environments were optimized by natural selection in response to the geomagnetic field. The relevant radical pair candidates outside of magnetoreception remain incompletely characterized, and the extent to which spin coherence persists under physiological conditions remains debated. In addition, ROS regulation emerges from numerous interacting biochemical pathways, making it difficult to isolate any specific magnetic contribution from other environmental and metabolic influences. Correlations between magnetic fields, latitude, and disease prevalence should therefore not be interpreted as evidence of causation. More generally, the paper does not claim that radical pair effects replace established biochemical mechanisms, but only that they may contribute as an additional layer of regulation. Future progress will require substantially more experimental work, including identification of biologically relevant radical pairs, direct measurements of magnetic field-dependent reaction yields in physiological systems, systematic perturbation of hyperfine environments through mutation or isotope substitution, and improved open-system models incorporating decoherence, temperature, and realistic cellular environments.

## Data Availability

The code used in this study is available from the corresponding authors upon reasonable request.
